# Biological and biochemical diversity in different biotypes of spotted stem borer, *Chilo partellus* (Swinhoe) in India

**DOI:** 10.1038/s41598-021-85457-2

**Published:** 2021-03-11

**Authors:** Mukesh K. Dhillon, Aditya K. Tanwar, Sandeep Kumar, Fazil Hasan, Suraj Sharma, Jagdish Jaba, Hari C. Sharma

**Affiliations:** 1 Division of Entomology, ICAR-Indian Agricultural Research Institute, New Delhi, 110012 India; 2 Biochemistry Laboratory, ICAR-National Bureau of Plant Genetic Resources, New Delhi, 110012 India; 3 International Crops Research Institute for the Semi-Arid Tropics, Patancheru, Telangana 502324 India

**Keywords:** Biochemistry, Ecology, Evolution, Zoology

## Abstract

Because of variation in incidence and severity of damage by *Chilo partellus* (Swinhoe) in different geographical regions, it is difficult to identify stable sources of resistance against this pest. Therefore, the present studies were undertaken on biological attributes (damage in resistant and susceptible genotypes, survival and development) and biochemical profiles (amino acids and lipophilic compound) of *C. partellus* populations from eight geographical regions to understand it’s population structure in India. There was a significant variation in biological attributes and biochemical profiles of *C. partellus* populations from different geographical regions. Based on virulence and biological attributes, similarity index placed the *C. partellus* populations in five groups. Likewise, lipophilic and amino acid profiling also placed the *C. partellus* populations in five groups. However, the different clusters based on biological and biochemical attributes did not include populations from the same regions. Similarity index based on virulence, biological attributes, and amino acids and lipophilic profiles placed the *C. partellus* populations in six groups. The *C. partellus* populations from Hisar, Hyderabad, Parbhani and Coimbatore were distinct from each other, indicating that there are four biotypes of *C. partellus* in India. The results suggested that sorghum and maize genotypes need to be tested against these four populations to identify stable sources of resistance. However, there is a need for further studies to establish the restriction in gene flow through molecular approaches across geographical regions to establish the distinctiveness of different biotypes of *C. partellus* in India.

## Introduction

There are distinct biological and genetic differences between geographically isolated populations of a given insect species as a result of diverse climatic conditions, variation in host plants, and their nutritional quality. Geographic isolation has been perceived to be one of the factors for phenological differentiation in the evolutionary history of herbivores, as the genetic exchange among the neighbouring populations is likely to be more frequent than among the populations separated geographically^1–3^. Wider geographic distribution also results in behavioural, physiological and genetic differences in insect populations^4–6^.

Spotted stem borer, *Chilo partellus* (Swinhoe) is one of the most widely distributed insect pests of coarse cereals in Asia and Africa^7^. The presence, abundance and intensity of infestation by *C. partellus* is influenced by both biotic and abiotic factors in a geographical region^7,8^. There is a wide physiological and behavioural variation in *C. partellus* populations in terms of diapause (hibernation in northern India and aestivation in southern India)^7,9^. The nature and intensity of diapause exercises a profound effect on post-diapause development and reproduction^10^, while the mating behaviour influences reproduction and population build-up of *C. partellus*^11^. Mating between adults from diapausing and nondiapausing *C. partellus* populations from different geographical regions also results in genetic polymorphism^12^, which will have implications to breed for resistance, and develop strategies for the management of this pest.

Genetic variation within a crop also results in differential herbivory by the insect pests in different geographical regions under diverse environmental conditions^13–17^. Matsubayashi et al.^18^ suggested that genetic variation is the basis for differences in host plant preference, and survival and development, which results in evolution of different biotypes of an insect. Information on geographical and molecular variation is important for understanding ecological speciation in phytophagous insects. Evolution of insect biotypes could be both allopatric or sympatric^19^. Several approaches such as mitochondrial DNA analysis, gene sequences, nested clade phylogeographic analyses (NCPA), and validation of NCPA have been advocated to understand genetic differentiation, which ascribe to evolutionary history of the insects^20–22^. Nuclear allozymes have also been used to understand the insect diversity and genetic structure of insect populations^23^.

The concerted research efforts over the past five decades have resulted in identification of several sources of resistance to *C. partellus* in sorghum and maize germplasm under natural infestation at a specific location or under artificial infestation with laboratory reared insects^24–27^. However, the sources identified as resistant/tolerant at one location sometimes exhibit a susceptible reaction at another location because of genotype × environment interactions, and/ or existence of genetically diverse populations of this pest in different geographic regions. The variation in virulence of different insect populations may have evolved due to long-term genetic differentiation and/or as a result of direct physiological response to host genotypes and the environment^6^. In nature, the existence of genetic variation within plants and herbivore communities influences the both virulence and biological attributes, resulting in evolutionary changes in insect population, which results in ecological speciation^18,28–31^. In addition to behavioural, physiological and molecular diversity, the information on biological performance and biochemical profiling is useful to understand the evolutionary changes in geographically isolated insect populations.

The amino acids and lipophilic compounds play a crucial role in metabolism and physiological processes in insect herbivores. However, there is little information on biological and biochemical variation in *C. partellus* populations from different geographical regions in India. Therefore, the present studies were undertaken to assess the variation in damage potential, biological attributes and biochemical profiles of *C. partellus* populations from different geographical regions infesting sorghum and maize in India. These studies will help to understand whether different biotypes of *C. partellus* exist in different regions in India, so as to develop appropriate strategies for evaluation of germplasm, breeding lines, mapping populations, and transgenic plants for resistance to this pest, and sustainable management of this pest.

## Results

### Variation in biological attributes of different *C. partellus* populations

There were significant differences in larval weights (F_8,32_ = 8.87; P < 0.001). The larval weights of Hisar, Coimbatore and Surat populations significantly greater than the populations from Delhi, Parbhani and Raichur (Table 1). The populations collected from Hisar, Parbhani and Raichur took significantly longer time to complete larval development as compared to the populations collected from Surat and Jhansi (F_8,32_ = 32.26; P < 0.001). The pupal period was significantly shorter in Jhansi and Coimbatore populations as compared to the populations collected from other geographical regions (F_8,32_ = 6.89; P < 0.001). The pupal weights were significantly lower in the populations collected from Delhi, Hisar, Hyderabad and Parbhani as compared to the Jhansi, Surat and Coimbatore populations (F_8,32_ = 24.41; P < 0.001). The males of insects collected from Hyderabad, and the laboratory population (F_8,32_ = 7.83; P < 0.001), and females of the Delhi population (F_8,32_ = 17.33; P < 0.001) lived for a significantly longer period as compared to the populations collected from other regions (Table 1).

**Table 1:** Biological performance of *Chilo partellus* populations from different geographical regions under laboratory conditions.

Population	Larval weight (mg/larva)	Larval period (days)	Pupal period (days)	Pupal weight (mg/pupa)	Male longevity (days)	Female longevity (days)
Delhi	81.8 ± 4.92a	26.0 ± 0.37b	8.7 ± 1.79b	65.0 ± 0.15a	4.1 ± 0.07a	6.3 ± 0.16c
Hisar	104.5 ± 1.66d	28.3 ± 0.11d	9.1 ± 1.77bc	64.4 ± 0.14a	4.3 ± 0.19ab	4.3 ± 0.18a
Jhansi	91.0 ± 2.77bc	24.3 ± 0.44a	7.8 ± 1.50a	89.7 ± 0.27d	4.3 ± 0.11ab	5.0 ± 0.01b
Parbhani	88.2 ± 2.03ab	29.3 ± 0.35d	8.7 ± 1.75b	67.8 ± 0.23ab	4.0 ± 0.32a	5.1 ± 0.30b
Raichur	88.9 ± 2.50ab	29.0 ± 0.42d	8.5 ± 0.46b	69.5 ± 0.21b	4.4 ± 0.18b	4.0 ± 0.01a
Surat	98.4 ± 3.27 cd	25.1 ± 0.08a	9.0 ± 2.73bc	77.7 ± 0.17c	3.6 ± 0.17a	4.4 ± 0.29a
Hyderabad	95.8 ± 0.70bc	27.6 ± 0.16c	8.9 ± 1.50bc	66.9 ± 0.27ab	5.1 ± 0.10c	4.1 ± 0.05a
Coimbatore	97.1 ± 2.43 cd	27.7 ± 0.19c	7.9 ± 1.63a	73.0 ± 0.21bc	3.8 ± 0.08a	4.4 ± 0.16a
Laboratory	80.5 ± 0.69a	26.7 ± 0.29b	9.4 ± 0.68c	69.7 ± 0.10b	4.8 ± 0.13bc	4.1 ± 0.10a

The values in a column following different letters are significant at P = 0.05 using post-hoc Tukey’s HSD test.

### Variation in damage potential and larval development of different *C. partellus* populations on sorghum and maize

There were significant differences in leaf damage due to different *C. partellus* populations on the stem borer-resistant sorghum genotype, IS 18551. Larval development and survival also varied between *C. partellus* populations from different geographical regions. The Hisar *C. partellus* population caused greater leaf damage (F_8,16_ = 6.08; P = 0.001), and exhibited better larval survival (F_8,16_ = 2.86; P = 0.035) as compared to other population, while the Raichur *C. partellus* population resulted in significantly more deadhearts (F_8,16_ = 4.56; P = 0.005) (Table 2). The larval weights were significantly greater (F_8,16_ = 4.87; P = 0.003) in Jhansi and Coimbatore populations as compared to the populations collected from other regions (Table 2).

**Table 2:** Damage due to *Chilo partellus* on resistant and susceptible sorghum and maize genotypes, and larval survival and weight of different geographical populations.

Population	Sorghum								Maize							
	Resistant cultivar				Susceptible cultivar				Resistant cultivar				Susceptible cultivar			
	LDR	DH	LS	LW	LDR	DH	LS	LW	LDR	DH	LS	LW	LDR	DH	LS	LW
Delhi	2.7b	24.6ab	26.7a	0.8ab	5.6a	82.4a	53.3a	2.8b	4.8bc	18.3a	56.7b	1.1a	5.7bc	35.9b	56.7a	2.4b
Hisar	3.6c	28.0b	43.3c	1.2bc	5.8a	80.6a	63.3a	2.7b	5.3c	30.1c	46.7b	2.3b	6.4c	33.9ab	63.3a	4.1d
Jhansi	2.1a	16.8a	36.7bc	1.5c	5.1a	81.1a	63.3a	2.8b	5.0bc	24.8b	33.3a	1.5b	4.5a	30.9ab	60.0a	2.6b
Parbhani	2.2ab	18.4a	23.3ab	0.5a	6.0a	78.8a	60.0a	2.5ab	5.0bc	22.7a	43.3ab	2.0b	5.1b	33.3ab	60.0a	4.0d
Raichur	2.0a	36.3c	23.3ab	0.7a	6.3a	79.5a	53.3a	2.7b	5.5c	22.5a	40.0a	1.7b	6.2c	37.2b	60.0a	3.1bc
Surat	1.9a	21.8ab	20.0a	0.8ab	6.3a	78.1a	93.3b	3.7c	4.4a	22.0a	40.0a	2.0b	5.4bc	27.6a	76.7b	4.3d
Hyderabad	2.5ab	23.9ab	30.0abc	0.7a	6.3a	82.9a	56.7a	1.8a	5.1bc	22.5a	30.0a	0.7a	5.0ab	28.6a	53.3a	1.5a
Coimbatore	2.8b	21.9ab	30.0abc	1.3c	6.3a	81.5a	86.7b	2.1ab	4.7ab	27.9bc	43.3ab	2.1b	6.4c	36.7b	90.0c	2.3b
Laboratory	2.6b	20.6ab	36.7bc	0.8a	6.5a	80.6a	53.3a	2.6b	4.2a	21.2a	53.3b	2.2b	4.0a	34.6b	60.0a	3.2c

*LDR* Leaf damage rating (1–9), *DH* Deadhearts (%), *LS* Larval survival (%), *LW* Larval weight (mg/larva). The values in a column following different letters are significant at P = 0.05 using post-hoc Tukey’s HSD test.

Leaf damage on the stem borer-susceptible sorghum genotype, Swarna varied significantly among *C. partellus* populations from different geographical regions (F_8,16_ = 1.25; P = 0.334). However, there were no significant differences in deadhearts caused by *C. partellus* populations from different geographical regions. The Surat and Coimbatore populations showed better larval survival on Swarna as compared to the populations from other regions (F_8,16_ = 10.30; P < 0.001). *Chilo partellus* larval weight was significantly greater (F_8,16_ = 5.05; P = 0.003) in the Surat population as compared to populations from other geographical regions (Table 2).

There were significant differences in leaf damage caused by *C. partellus* populations from different geographical regions on the stem borer-resistant maize genotype, CPM 15. Larval development and survival also varied between *C. partellus* populations from different geographical regions. The Hisar and Raichur populations caused greater leaf damage (F_8,16_ = 2.71; P = 0.043), while the Hisar and Coimbatore populations caused more deadhearts (F_8,16_ = 6.69; P < 0.001) as compared to the populations collected from other regions (Table 2). The larval survival was better in Delhi, Hisar, Parbhani, Coimbatore and laboratory populations (F_8,12_ = 2.66; P = 0.046) as compared to the populations collected from other regions (Table 2). The larval weights were significantly greater (F_8,32_ = 5.89; P = 0.001) in Delhi and Hyderabad populations as compared to populations collected from other geographical regions (Table 2).

There were significant differences in leaf damage caused by different *C. partellus* populations on the stem borer-susceptible maize genotype, Basi Local. Larval development and survival also varied significantly across *C. partellus* populations from different geographical regions. The Jhansi, Hyderabad and laboratory populations caused lower leaf damage (F_8,16_ = 7.09; P < 0.001), while the Surat and Hyderabad populations caused lower deadhearts (F_8,16_ = 3.37; P = 0.018) as compared to populations from other regions (Table 2). The Surat and Coimbatore populations showed better larval survival (F_8,12_ = 3.89; P = 0.010) on the Basi Local maize genotype as compared to other populations (Table 2). *Chilo partellus* larval weights were significantly greater (F_8,32_ = 15.89; P < 0.001) in the Hisar, Parbhani and Surat populations as compared to populations collected from other geographical regions (Table 2).

### Variation in lipophilic compounds in the larvae of different *C. partellus* populations

A total of 26 lipophilic compounds were detected in different *C. partellus* populations (Supplementary Fig. 1). There were significant differences in the amounts of different lipophilic compounds in the larvae of *C. partellus* populations from different geographical regions in India (Table 3). The amounts of palmitoleic acid (F_8,16_ = 279.55; P < 0.001), palmitic acid (F_8,16_ = 86.87; P < 0.001) and oleic acid (F_8,16_ = 16.96; P < 0.001) were significantly lower, while those of n-pentadecanol (F_8,16_ = 12.72; P < 0.001), 1-octadecanol (F_8,16_ = 17.28; P < 0.001), 1-nonadecene (F_8,16_ = 33.39; P < 0.001), margaric acid (F_8,16_ = 254.52; P < 0.001), 9-octadecen-1-ol (F_8,16_ = 28.54; P < 0.001), methyl 11-eicosenoate (F_8,16_ = 12.99; P < 0.001), eicosanoic acid (F_8,16_ = 5.75; P = 0.001), 1,16-hexadecanediol (F_8,16_ = 41.50; P < 0.001), erucic acid (F_8,16_ = 12.61; P < 0.001), (Z)-14-tricosenyl formate (F_8,16_ = 3.19; P = 0.003), squalene (F_8,16_ = 35.49; P < 0.001), 1-triacontanol (F_8,16_ = 3.22; P = 0.002), cholesterol (F_8,16_ = 113.71; P < 0.001), gamma-ergostenol (F_8,16_ = 3.32; P = 0.020), chondrillasterol (F_8,16_ = 3.03; P = 0.008) and lathosterol (F_8,16_ = 8.57; P < 0.001) were significantly greater in the larvae of Hisar and laboratory populations (except eicosanoic acid) as compared to the populations collected from other geographical regions (Table 3). Greater amount of palmitoleic acid (F_8,16_ = 279.55; P < 0.001) was recorded in the Delhi population, while the amounts of methyl 3-methxytetradecanoate (F_8,16_ = 15.67; P < 0.001), methyl 14-methxyhexadecanoate (F_8,16_ = 3.25; P = 0.021), linoleic acid (F_8,16_ = 14.39; P < 0.001) and oleic acid were greater in Parbhani population; l-(+)-ascorbic acid 2,6-dihexadecanoate (F_8,16_ = 128.15; P < 0.001) in Raichur and Coimbatore populations; stearic acid in Surat population; and myristic acid (F_8,16_ = 53.66; P < 0.001) and stearic acid (F_8,16_ = 14.32; P < 0.001) in Hyderabad population as compared to *C. partellus* populations from other geographical regions (Table 3). However, the amount of methyl 16-methyl-heptadecanoate (F_8,16_ = 5.86; P = 0.001) was significantly lower in the Surat and Hyderabad populations as compared to other geographical *C. partellus* populations (Table 3).

**Table 3:** Lipophilic content per compound in *Chilo partellus* larvae from different geographical regions of India.

Lipophilic compounds	Lipophilic content (%)								
	Delhi	Hisar	Jhansi	Parbhani	Raichur	Surat	Hyderabad	Coimbatore	Laboratory
n-Pentadecanol	0.40a	1.27b	0.52a	0.68a	0.53a	0.44a	0.57a	0.44a	1.54b
Methyl 3-methoxytetradecanoate	0.72ab	1.11c	0.56a	1.59d	1.08c	0.62a	0.67a	0.91b	1.63d
Myristic acid	0.51d	0.17a	0.39b	0.54d	0.44c	0.47c	0.63e	0.51d	0.46c
1-Octadecanol	0.45a	1.03b	0.55a	0.86a	0.61a	0.47a	0.67a	0.51a	1.40b
Palmitoleic acid	4.07e	2.27a	2.96b	3.31c	3.71d	3.02b	2.91d	3.68d	2.55a
Palmitic acid	22.30b	18.96a	23.41b	21.80b	22.32b	23.53b	23.83b	21.73b	18.22a
l-(+)-Ascorbic acid 2,6-dihexadecanoate	19.38c	15.33b	10.12a	9.75a	21.98d	18.98c	15.17b	21.72d	15.03b
1-Nonadecene	0.24a	1.22b	0.32a	0.49a	0.33a	0.27a	0.39a	0.28a	1.47b
Methyl 14-methylhexadecanoate	0.03a	0.13b	0.04a	0.29c	0.05a	0.03a	0.05a	0.04a	0.05a
Margaric acid	0.09b	0.37e	0.08b	0.10bc	0.05a	0.11c	0.14d	0.06a	0.13d
Methyl 16-methyl-heptadecanoate	11.80b	9.82ab	13.48b	11.03ab	10.23ab	6.77a	6.22a	11.82b	12.05b
Linoleic acid	11.79a	11.82a	13.46bc	15.35c	10.22a	14.37bc	15.03c	11.79a	12.05b
Oleic acid	19.65ab	17.00a	26.86c	26.06c	20.93b	22.19b	24.32c	18.78a	18.16a
Stearic acid	3.42b	2.39a	4.26c	3.67b	4.19bc	5.47d	5.26d	3.88bc	3.59b
9-Octadecen-1-ol	0.03a	0.29c	0.02a	0.05a	0.01a	0.04a	0.04a	0.01a	0.20b
Methyl 11-eicosenoate	0.09a	1.18c	0.11a	0.25a	0.11a	0.12a	0.27a	0.14a	0.69b
Eicosanoic acid	0.22a	0.73b	0.22a	0.21a	0.21a	0.28a	0.38a	0.26a	0.28a
1,16-Hexadecanediol	0.09ab	0.55c	0.02a	0.10ab	0.02a	0.02a	0.02a	0.02a	0.13b
Erucic acid	0.36a	1.77c	0.31a	0.63ab	0.25a	0.37a	0.45a	0.62ab	1.02b
(Z)-14-Tricosenyl formate	0.04a	1.27b	0.03a	0.09a	0.02a	0.12a	0.02a	0.04a	0.72b
Squalene	0.27a	1.00b	0.16a	0.23a	0.18a	0.23a	0.22a	0.22a	1.01b
1-Triacontanol	0.11a	1.70b	0.03a	0.03a	0.11a	0.04a	0.02a	0.10a	1.15b
Cholesterol	1.67a	3.82c	1.68a	1.41a	1.46a	1.61a	2.54b	1.48a	2.86b
Gamma.-Ergostenol	0.02a	1.49b	0.02a	0.07a	0.11a	0.02a	0.01a	0.13a	1.00b
Chondrillasterol	1.31c	2.21d	0.15a	0.91bc	0.75b	0.38ab	0.09a	0.30a	1.58c
Lathosterol	0.94c	1.10c	0.24a	0.50b	0.10a	0.03a	0.08a	0.53b	1.03c

The values in a row following different letters are significant at P = 0.05 using post-hoc Tukey’s HSD test.

### Variation in amino acids in different *C. partellus* populations

The amino acid profiling separated 17 amino acids across the test populations (Supplementary Fig. 2). The larvae of Coimbatore and laboratory populations had significantly lower amounts of serine (F_8,16_ = 11.61; P < 0.001), glutamic acid (F_8,16_ = 6.75; P < 0.001), histidine (F_8,16_ = 26,906.1; P = 10.46), threonine (F_8,16_ = 5.03; P = 0.003), proline (F_8,16_ = 6.91; P < 0.001), cystine (F_8,16_ = 2.70; P = 0.043), tyrosine (F_8,16_ = 6.96; P < 0.001), valine (F_8,16_ = 6.67; P < 0.001), methionine (F_8,16_ = 4.50; P = 0.005), isoleucine F_8,16_ = 3.84; P = 0.011), leucine (F_8,16_ = 4.74; P = 0.004), and phenylalanine (F_8,16_ = 5.35; P = 0.002), while Parbhani population had greater amounts of these amino acids (except cystine) and that of glycine (F_8,16_ = 8.07; P < 0.001), arginine (F_8,16_ = 15.15; P < 0.001) and arginine (F_8,16_ = 15.15; P < 0.001) as compared to other geographical *C. partellus* populations (Table 4). The amount of aspartic acid (F_8,16_ = 25.77; P < 0.001) was greater in Coimbatore population, while the amount of lysineHCL (F_8,16_ = 3.60; P = 0.014) was greater in Jhansi and Hyderabad populations as compared to other geographical populations (Table 4). The Delhi, Hisar, Jhansi, Raichur, Surat and Hyderabad populations had moderate amounts of these amino acids, although there were a few exceptions (Table 4).

**Table 4:** Amino acid amount in *Chilo partellus* larvae from different geographical regions of India.

Amino acids	Amino acid amount (ug/100 mg)								
	Delhi	Hisar	Jhansi	Parbhani	Raichur	Surat	Hyderabad	Coimbatore	Laboratory
Aspartic acid	30.78b	24.35ab	25.80ab	28.73ab	25.33ab	44.99c	23.39a	54.56d	21.27a
Serine	28.78bc	37.18c	23.98a	48.97d	32.59b	27.10b	29.37b	18.15a	24.42a
Glutamic acid	31.63a	40.70b	42.18b	58.36c	33.57ab	29.51a	30.71ab	21.42a	23.41a
Glycine	33.24bc	43.84d	23.17ab	46.83d	37.47 cd	27.73bc	37.03 cd	12.82a	25.25b
Histidine	37.04b	50.05 cd	24.63a	61.12d	42.05bc	38.70b	54.00 cd	21.83a	32.13ab
Arginine	46.78bc	67.44d	32.52ab	88.06e	54.15 cd	45.32bc	69.22d	25.98a	43.99bc
Threonine	25.28ab	30.58b	23.28a	42.53c	36.13bc	24.47a	27.67b	15.99a	19.39a
Alanine	21.20bc	25.62c	21.34b	27.66c	23.94c	16.51b	24.51c	7.90a	15.51b
Proline	23.33b	30.43c	20.86b	33.57c	30.03c	20.65b	28.78c	9.93a	15.73ab
Cystine	3.34a	6.17b	2.20a	5.96ab	6.21b	3.52a	9.29b	4.26a	4.29a
Tyrosine	88.08ab	89.95ab	79.44a	153.97c	122.52bc	109.24b	126.05bc	55.00a	53.94a
Valine	20.36b	27.05c	21.59b	34.89d	26.45c	18.86a	21.52b	11.47a	14.24ab
Methionine	169.10b	190.80bc	132.70ab	243.00c	242.10c	158.60ab	272.70c	66.40a	116.70a
LysineHCl	6.36a	8.84ab	15.62b	4.98a	9.40ab	3.73a	12.84b	2.65a	3.67a
Isoleucine	14.40a	20.03b	16.09ab	22.72b	23.46b	13.57a	22.48b	8.20a	11.08a
Leucine	26.73b	33.34bc	28.01b	36.98c	40.35c	23.99ab	31.97bc	11.71a	17.51a
Phenylalanine	52.84bc	57.30bc	40.80ab	86.91d	68.72 cd	53.42bc	65.71 cd	30.62a	41.06ab

The values in a row following different letters are significant at P = 0.05 using post-hoc Tukey’s HSD test.

### Biological and biochemical trait-based guilds of *C. partellus* populations

**Figure 1:**
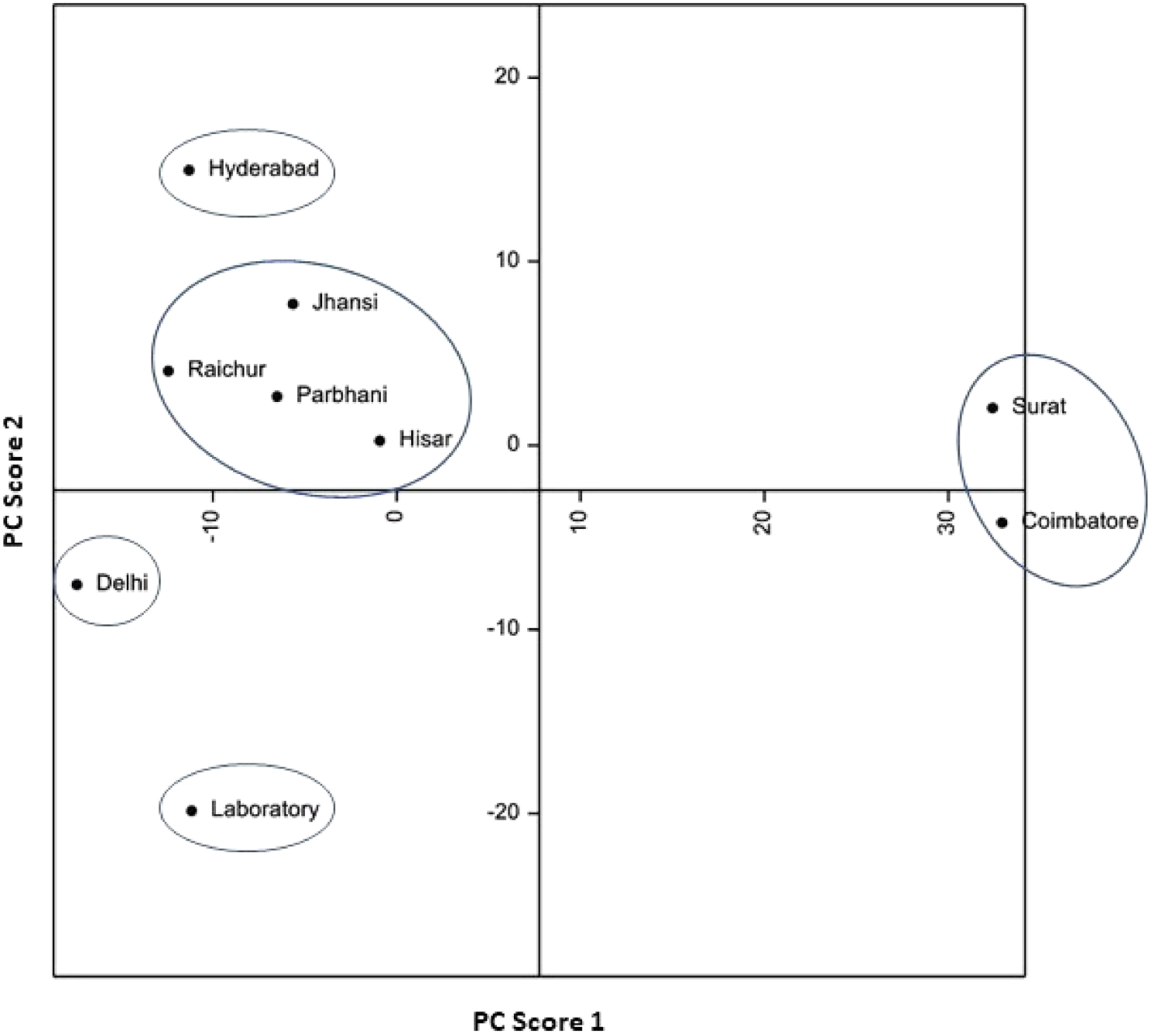
Diversity in different geographical *C. partellus* populations based on biological traits, and damage caused to sorghum and maize.

**Figure 2:**
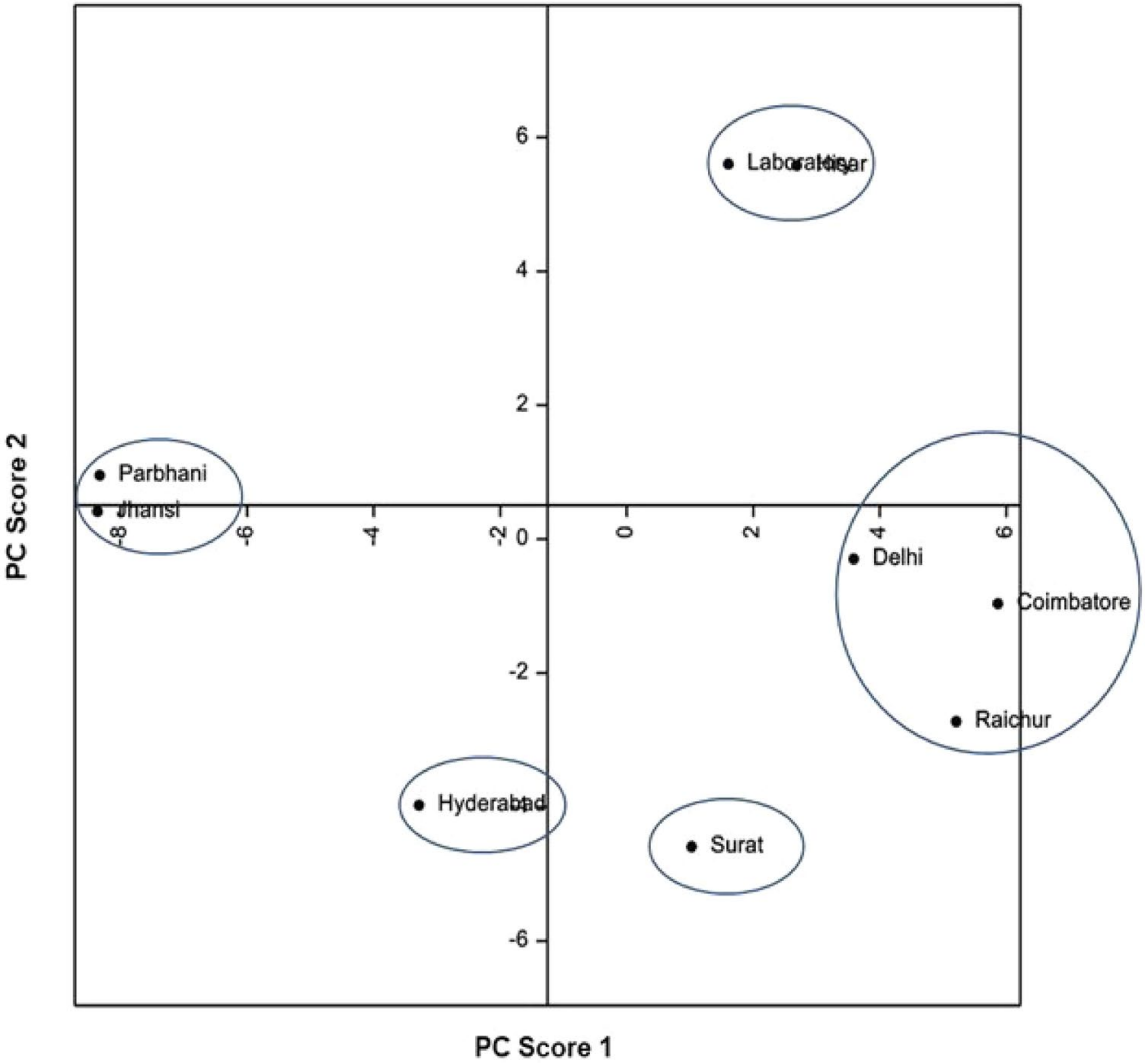
Diversity in different geographical *C. partellus* populations based on lipophilic compounds.

**Figure 3:**
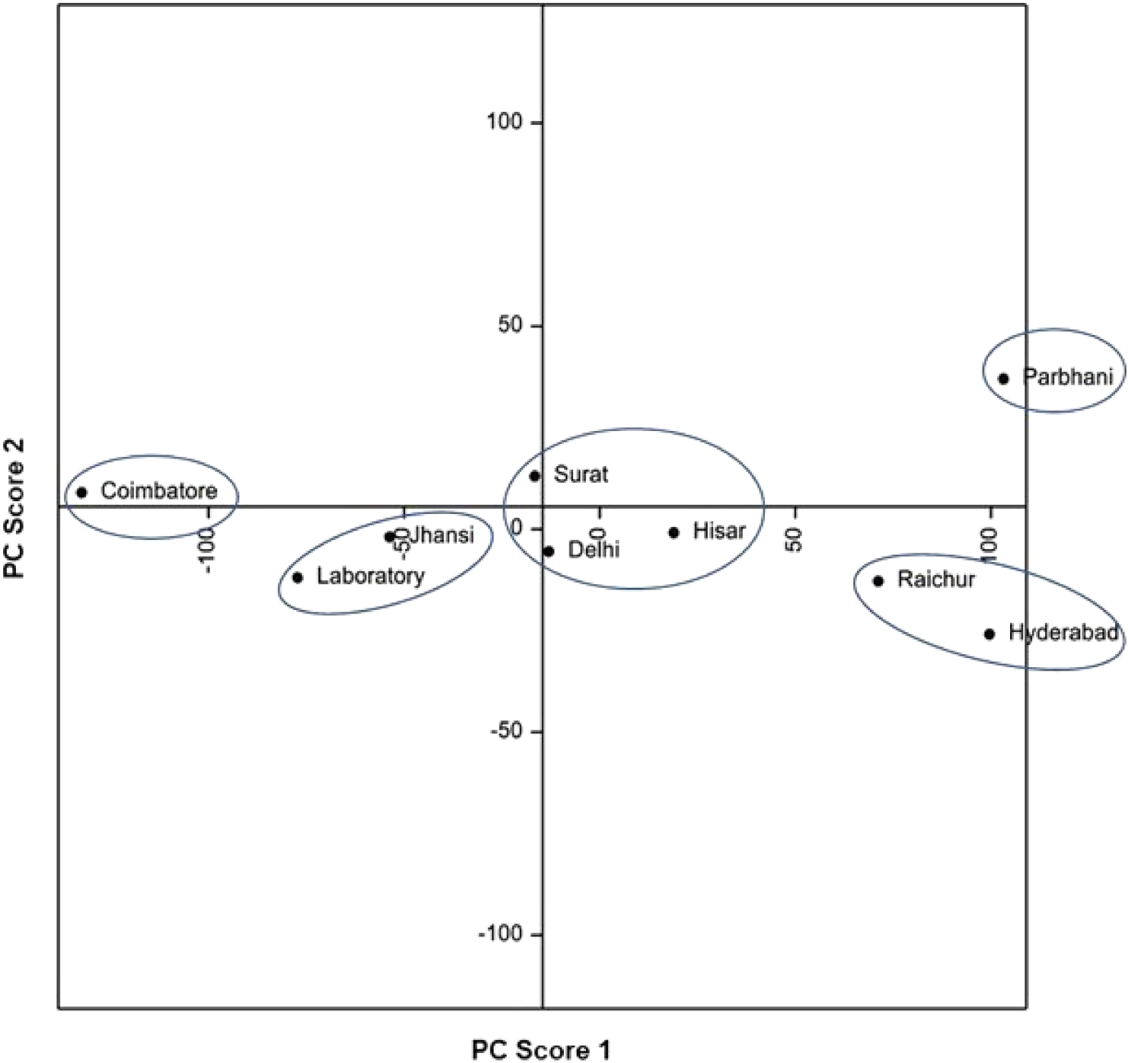
Diversity in different geographical *C. partellus* populations based on amino acids.

**Figure 4:**
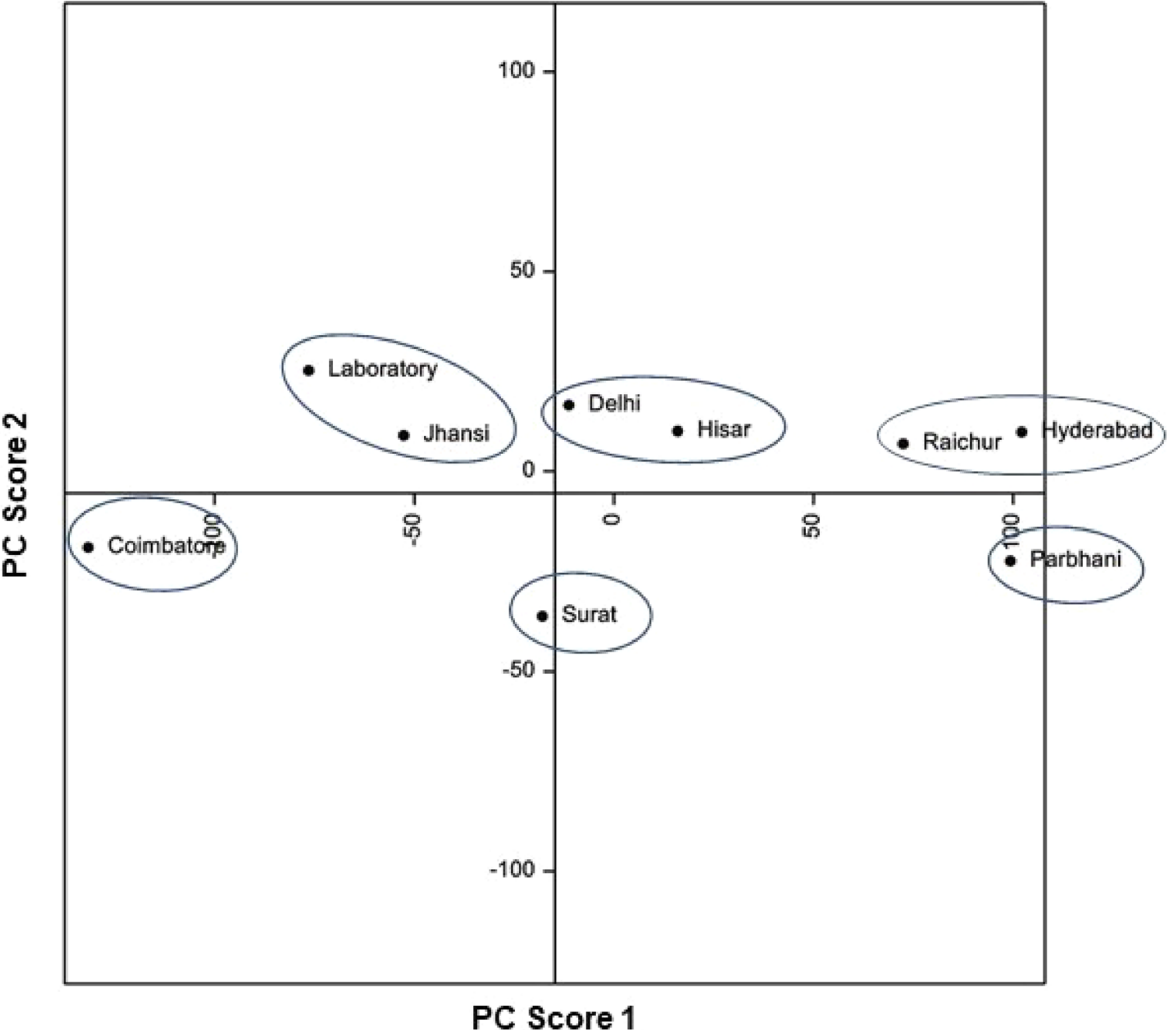
Diversity in different geographical *C. partellus* populations based on lipophilic compounds, amino acids, biological traits, and damage caused to sorghum and maize.

Principal component analyses based on biological attributes, damage potential and survival on sorghum and maize, and lipophilic compounds and amino acid profiles, indicated considerable diversity among *C. partellus* populations from different geographical regions. Principal component analysis based on biological attributes, damage potential and survival placed the *C. partellus* populations into V groups [I = Surat and Coimbatore; II = Jhansi, Raichur, Parbhani and Hisar; III = Hisar; IV = Delhi; and V = Laboratory] (Fig. 1). Based on lipophilic compounds, the stem borer populations were placed into V groups [I = Hisar and laboratory; II = Parbhani and Jhansi; III = Hyderabad; IV = Surat; and V = Delhi, Coimbatore and Raichur] (Fig. 2). The amino acid profiling also placed the stem borer populations in V groups [I = Parbhani; II = Surat, Delhi and Hisar; III = Jhansi and laboratory populations; IV = Coimbatore; and V = Raichur and Hyderabad] (Fig. 3). However, the grouping based on both biological and biochemical parameters placed the *C. partellus* populations in VI groups [I = Raichur and Hyderabad; II = Delhi and Hisar; III = Jhansi and laboratory populations; IV = Coimbatore; V = Surat; and VI = Parbhani] (Fig. 4). Individual as well as pooled parameters placed Hisar, Hyderabad, Parbhani and Coimbatore populations into diverse groups, thus indicating presence of at least four different ecotypes/biotypes of *C. partellus* in India.

## Discussion

Environmental factors influence behavior and biology of insects, plant growth, and biochemical composition of both insect and the host plant, which exercises a considerable influence on insect–host plant interactions. Genotype x environment interactions are a major constraint in screening and breeding for resistance to insect pests^32^. Occurrence of new biotypes or changes in the genotypic expression of resistance to insects as a result of climate change may limit the use of certain insect-resistant varieties in crop improvement^33^, which necessitates multilocational testing of the identified sources and breeding lines to identify stable and diverse sources of resistance or establish the presence of new insect biotypes^34^. Considerable progress has been made over the five decades in introgressing genes from diverse sources to develop varieties with resistance to the target insect pests^32,34^. Use of molecular techniques for identification and utilization of insect resistance, understanding the nature of gene action and metabolic pathways is important for gaining a better understanding of the nature and inheritance of resistance to insect pests. However, usefulness and adoption of biotechnological approaches will depend on developing a full understanding of the interaction of genes within their genomic environment, and with the environment in which their conferred phenotype interacts^35^.

Geographic isolation acts as barrier for gene flow between insect populations within a species, and thus, lead to ecological speciation or emergence of new strains/biotypes^36–39^. Geographical isolation and genetic variation within host plants triggers behavioral and physiological changes in insect populations, which ultimately may lead to ecological speciation. Because of the distinct behavior of the spotted stem borer, *C. partellus* in northern (diapausing population) and southern (aestivating population) India, it is important to understand the differences in biological and biochemical attributes of stem borer populations from different geographical regions in relation to expression of genotypic resistance to this pest in its principal host plants—sorghum and maize. The present studies revealed significant differences in damage potential and biological attributes of *C. partellus* populations from different geographical regions. The *C. partellus* populations from Delhi, Hisar, Hyderabad and Coimbatore were quite distinct in terms of their damage potential and biological attributes on resistant and susceptible genotypes of sorghum and maize. These differences in *C. partellus* populations may be due to variation in climatic conditions such as temperature and photoperiod, and the changes in morphological and biochemical attributes of maize and sorghum as a result of changes in climatic conditions, resulting in distinct insect–host plant interactions. Genetic variation has earlier been reported in different geographic strains of rice stem borer, *Chilo suppressalis* (Walker) collected from diverse rice genotypes^40^. Extensive phenotypic plasticity has been observed in European species of dung flies, *Scathophaga stercoraria* (L.) and *Sepsis cynipsea* (L.) populations collected from high and low altitudes^5^. However, altitudes alone were not responsible for variation in phenology, body size and genetic adaptation, and hence other geographic variables may also be responsible for the genotypic and phenotypic variation among these populations.

Nuclear allozymes have been used to decipher the genetic structure of European corn borer, *Ostrinia nubilalis* (Hub.) populations from different host plants^23^. Allozymes have also been used for mapping genetic variation in different geographical populations of the Brazilian phlebotomine sand fly, *Lutzomyia longipalpis* (Lutz and Neiva)^41^. Using host plant differentials (resistant and susceptible genotypes) of rice, at least seven distinct biotypes of Asian rice gall midge, *Orseolia oryzae* (Wood-Mason) have been identified in India^42^. Significant progress has also been made in identifying, tagging and pyramiding the genes conferring resistance to different rice gall midge biotypes using marker assisted selection to develop midge-resistant rice varieties^43^. Similarly, the avirulent and virulent response of different Hessian fly, *Mayetiola destructor* (Say) populations in USA in wheat cultivars or germplasm lines have led to identification of several biotypes and the resistance genes to develop Hessian fly-resistant wheat varieties^44,45^.

In the present studies, the amino acid profiling showed significant differences in different *C. partellus* populations, and distinguished Coimbatore, Parbhani, Jhansi, Hisar and Hyderabad populations; while lipophilic profiling distinguished Hisar, Parbhani, Surat, Hyderabad and Coimbatore populations from each other. The results suggested that biological attributes and biochemical profiles are equally effective in distinguishing different stem borer populations, and can be used to identify different populations of a given insect species. The amino acid and lipophilic profiling, in addition to distinguishing different geographical populations, is also useful to understand the role of these biomolecules in host plant resistance to *C. partellus*^46,47^.

Biotype framework has contributed significantly in crop improvement programs for resistance to insect pests, such as the case of Asian rice gall midge, *Orseolia oryzae* (Wood-Mason)^48^. However, there are instances where the biotype framework has failed to contribute to breeding and resistance deployment programs, such as the case of brown planthopper, *Nilaparvata lugens* (Stal)^49^. Utility of variability or biotype concept in pest management springs out of the conceptual vagueness of its evolutionary mechanism^50^. Some of the variation in response to a pest control tactic could be due to phenotypic plasticity, endosymbionts, geographic race, host race and/ or a different species. The use of term biotype in pest management is quite contentious^50–52^. Failure of a control tactic, particularly breakdown of resistance to a particular insect population, which has apparently adapted to a particular host or cultivar, is often considered to be a new or distinct entity, and given the non-formal category ‘biotype’. However, the variation in insect response could be due to nongenetic polyphenism, polygenic variation within populations, geographic races, host races, and/or species^50^.

Molecular studies have provided evidence for restricted or absence of gene flow to establish fixed differences or strongly supported clades, indicating existence of races to designate insect biotypes in addition to response to management tactics^53^. Some of these categories can be tested by examining the population genetic structure of the target insect species^54^. However, comparative population genetic analysis of virulent and avirulent (i.e., unable to feed on resistant cultivars) biotypes of soybean aphid, *Aphis glycines* Matsumura has shown that these populations are genetically indistinguishable across biotypes, with high rates of inter-population admixture. Therefore, there is a need for in-depth studies on the genetic structure of different geographical populations of *C. partellus* to establish that there are distinct overlapping populations of this insect in India.

Damage potential, biological attributes, and lipophilic and amino acid profiling exhibited considerable diversity in the stem borer populations, and indicated that Hisar, Hyderabad, Parbhani and Coimbatore populations were quite distinct, suggesting that there are at least four different biotypes of *C. partellus* in India. However, population differentiation requires not just differentiation in the phenotype of host performance (the loci that confer greater fitness on a host) which might provide better capability to adapt to a particular host, but also the evidence for persistent restriction of gene flow across host associated populations or geographical regions. Therefore, there is also a need for further studies to establish the restriction in gene flow in *C. partellus* through molecular approaches across geographical regions, as cultivation of the host plants and the weather conditions gradually change, and overlap from North to South India.

## Materials and methods

### Collection and maintenance of different geographical populations of *C. partellus*

**Figure 5:**
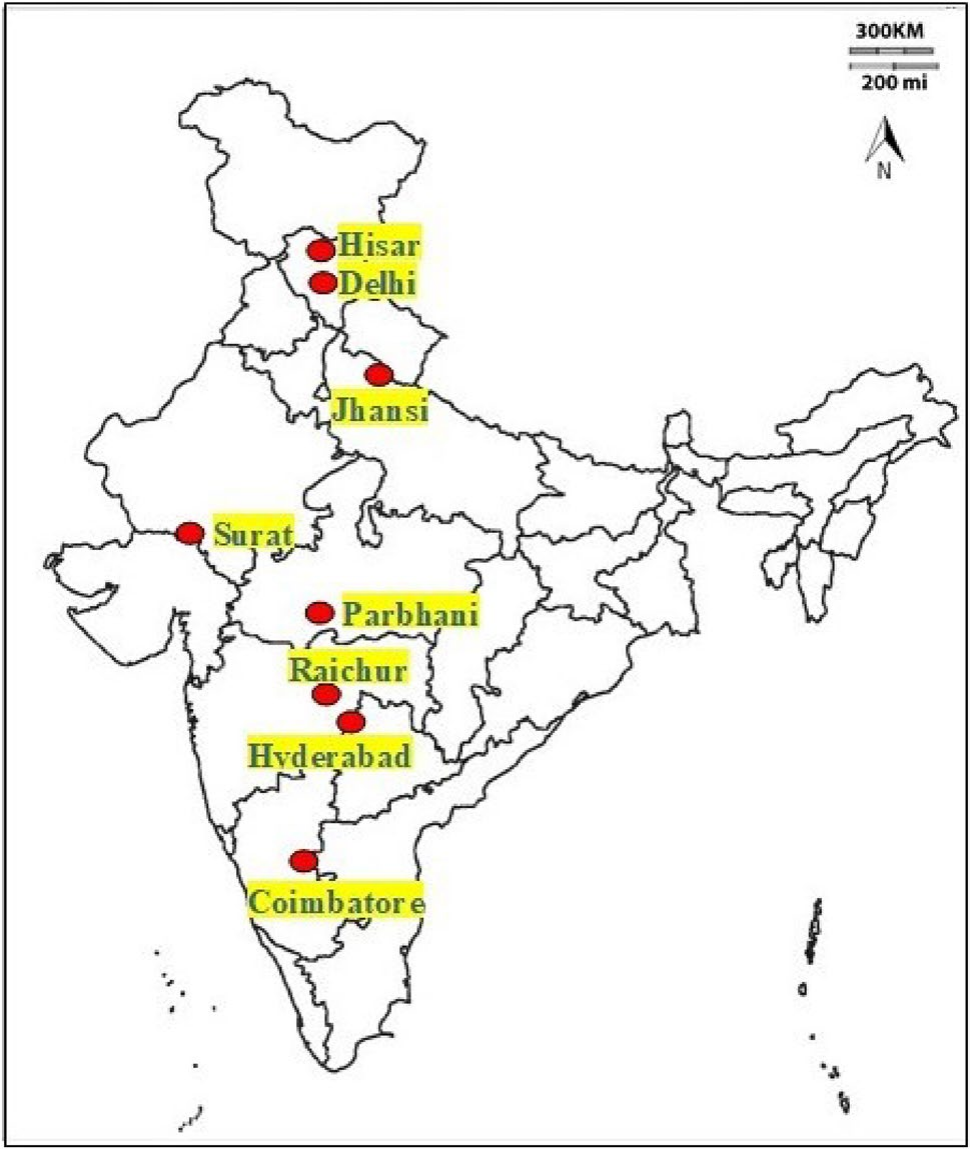
Map depicting geographic locations from where *C. partellus* larvae were collected.

The spotted stem borer, *C. partellus* larvae were collected from maize and sorghum in different geographical regions in India, i.e., Delhi (28.6139° N, 77.2090° E; AMSL: 216 m), Hisar (29.1492° N, 75.7217° E; AMSL: 215 m), Jhansi (25.4484° N, 78.5685° E; AMSL: 285 m), Surat (21.1702° N, 72.8311° E; AMSL: 13 m), Parbhani (19.2610° N, 76.7767° E; AMSL: 347 m), Hyderabad (17.3850° N, 78.4867° E; AMSL: 505 m), Raichur (16.2120° N, 77.3439° E; AMSL: 407 m) and Coimbatore (11.0168° N, 76.9558° E; AMSL: 411 m) (Fig. 5). The field collected *C. partellus* populations were brought to Indian Council of Agricultural Research-Indian Agricultural Research Institute (ICAR-IARI), New Delhi, India, and reared separately on green maize stalks under laboratory conditions at 27 ± 1 °C, 65 ± 5% RH, and 12 L: 12 D till pupation. Adults emerged from these populations were released in oviposition cages. The oviposition cages were covered with wax-paper from outside to serve as oviposition substrate. The wax-papers were changed daily, and the papers with eggs were kept at 27 ± 1 °C for hatching and use in different experiments.

The field collected populations along with the laboratory-maintained *C. partellus* culture (initially collected from Hisar and completed 10 generations under laboratory conditions) at the Division of Entomology, ICAR-IARI, New Delhi, India, were used for assessing the damage on different genotypes of sorghum and maize, and biological and biochemical diversity of *C. partellus*.

### Biological performance of different *C. partellus* populations

The F_1_ generation neonate larvae of the above-mentioned field collected populations, and those from laboratory-maintained *C. partellus* culture were used to study the variation in biological attributes in the laboratory at 27 ± 1 °C, 65 ± 5% RH, and 12 L: 12D. The populations of field collected and laboratory insects were all reared on artificial diet^55^. For this purpose, 200 ml artificial diet was poured into plastic jars (500 ml capacity) having lids fitted with wire-mesh, and allowed to settle for 4 h. Fifty neonate *C. partellus* larvae were released in each jar, and there were five replicates for each population in a completely randomized design. After releasing the larvae in the artificial diet, the jars were kept in the dark for 3-days to allow the larvae to settle on the artificial diet. Twenty days after inoculation, each larva (4th instar stage) was weighed after starvation for 4-h on an electronic balance (Contech, CB-120), and the weights recorded as mg/larva. Each jar was observed daily for formation of pupae. The period between the date of releasing the larvae to the artificial diet till the date of pupation was calculated as the larval period; from the date of pupation to the date of adult emergence was considered as the pupal period. Pupal weights were recorded on electronic balance (Contech, CB-120) for each pupa separately, one day after pupation, and data expressed as mg/pupa. The adults emerging from each jar were separated into males and females, and kept separately in oviposition cages. The longevity of males and females was recorded separately, averaged per replication and expressed in days. In addition to insects used for measuring biological parameters, each population was also multiplied for conducting field and laboratory bioassays, and profiling of amino acids and lipophilic compounds. In that case, third-instar *C. partellus* larvae (weighing around 50 mg) were collected, starved for 4 h, and stored in glass vials at – 20 °C for estimation of amino acids and lipophilic compounds.

### Damage and larval development of different *C. partellus* populations on sorghum and maize

Two genotypes each of maize (resistant: CPM 15; susceptible: Basi Local) and sorghum (resistant: IS 18551; susceptible: Swarna) were sown each in two rows of 4 m row length in a randomized complete block design, and there were three replications for each aforesaid *C. partellus* population. The test maize and sorghum genotypes were covered with a plot cage comprising of iron pipes (6 m length × 5 m width × 2.7 m height) clamped together to make the structure and covered with fine mosquito net restricting the in and out movement of *C. partellus*. Fifteen days old seedlings of the test maize and sorghum genotypes were used for laboratory and field studies. For laboratory studies, three leaf discs (5 cm dia.) from the 3rd leaf of each genotype were prepared, and inoculated with 10 neonate *C. partellus* larvae from each population. The laboratory bioassays were conducted in growth chambers at 27 ± 2 °C, 70 ± 5% RH, and a photoperiod of 12:12 (L:D). After 5-days of larval infestation, observations were recorded on larval weight (mg/larva) and larval survival (%). For field studies, each plant of test maize and sorghum genotypes was infested with 5 neonate *C. partellus* larvae from each population in the designated plots. Two weeks after larval inoculation, observations were recorded on leaf damage rating on a scale of 1 to 9 (1 ≤ 10% leaf area damaged and 9 ≥ 80% leaf area damaged). Plants with deadhearts were recorded three weeks after infestation, and expressed as a percentage of the total number of plants.

### Estimation of lipophilic compounds in *C. partellus* larvae from different populations

The lipophilic compounds in the F_1_ *C. partellus* larvae from different geographical populations were estimated by using gas chromatography–mass spectroscopy (GC–MS) as described by Kumar and Dhillon^56^. Three *C. partellus* larvae per population (making three replications) were processed, and the fatty acids were converted to their respective methyl esters. The GCMS-QP2010 Ultra system with autosampler AOC-20i (Shimadzu, Japan) was used for separation/estimation of lipophilic compounds. The chromatograms and mass spectra were analysed using the Labsolutions GCMS software version 2.71 (Shimadzu, Japan). The lipophilic compounds were identified using MS libraries (NIST08, Wiley8). The fatty acids were also verified using NIST confirmed fatty acid methyl ester standards (99.9%) obtained from SUPELCO Analytical, Bellefonte, PA, USA.

### Estimation of amino acids in *C. partellus* larvae from different populations

The amino acids in the F_1_ *C. partellus* larvae of different geographical populations were estimated using high performance liquid chromatography-photodiode array detector (HPLC–PDA) method described by Dhillon et al.^57^. Three *C. partellus* larvae per population (making three replications) were processed and derivatized with AccQ-Fluor reagent kit (WAT052880-Waters Corporation, USA), separated on a Waters 2707 Module HPLC System attached to a PDA (Model PDA 2998), and detected using PDA at 254 nm. The amino acid peaks were acquired by using Empower Pro Software® by Waters Corporation (2005–2008), and their amounts calculated based on amino acid calibration standards (Thermo Scientific Amino Acid Standard H, Prod # NCI0180), and expressed as µg/100 mg.

### Statistical analysis

The data on biological attributes and damage potential in different host crops, and amino acid and lipophilic compounds in the larvae of different *C. partellus* populations were subjected to analysis of variance (ANOVA). The significance of differences was judged by *F*-test, and the treatment means were compared using post-hoc Tukey’s HSD test carried out by statistical software SPSS®. The diversity in different geographical *C. partellus* populations based on biological attributes, damage potential in different host crops, amino acids and lipophilic compounds were determined by using principal component analysis.

## Supplementary Information

Supplementary Information

## References

[1] Dujardin JP, Guhl F (1997). Aporte de la genetica poblacional al control y vigilancia de vectores de la enfermedad de Chagas. Curso Posgrado Genética Poblacional de Triatomineos Aplicada al Control Vectorial de la Enfermedad de Chagas.

[2] Pires HHR, Barbosa SE, Margonari C, Jurberg J, Diotaiuti L (1998). Variations of the external male genitalia in three populations of *Triatoma infestans* Klug, 1834. Minist. Saúde.

[3] Bambou AE, Ouantinam SFB, Thiaw C, Mokossesse JA, Ndong A, Kane M, Sembène M (2014). Comparing genetic diversity of *Sitophilus zeamais*(Motchulsky) populations sampled in several agro-ecological areas between Central African Republic and Senegal. South Asian J. Exp. Biol..

[4] Baldwin JD, Dingle H (1986). Geographic variation in the effects of temperature on life-history traits in the large milkweed bug *Oncopeltus fasciatus*. Oecologia.

[5] Blanckenhorn WU (1997). Altitudinal life history variation in the dung flies *Scathophaga stercoraria* and *Sepsis cynipsea*. Oecologia.

[6] Ikten C, Skoda SR, Hunt TE, Molina-Ochoa J, Foster JE (2011). Genetic variation and inheritance of diapause induction in two distinct voltine ecotypes of *Ostrinia nubilalis* (Lepidoptera: Crambidae). Ann. Entomol. Soc. Am..

[7] Dhillon MK, Hasan F, Tanwar AK, Bhadauriya APS (2017). Effects of thermo-photoperiod on induction and termination of hibernation in *Chilo partellus* (Swinhoe). Bull. Entomol. Res..

[8] Sharma HC (1993). Host plant resistance to insects in sorghum and its role in integrated pest management. Crop Prot..

[9] Dhillon MK, Hasan F, Tanwar AK, Bhadauriya APS (2019). Factors responsible for aestivation in spotted stem borer, *Chilo partellus* (Swinhoe). J. Exp. Zool. A.

[10] Dhillon MK, Hasan F (2018). Consequences of diapause on post-diapause development, reproductive physiology and population growth of *Chilo partellus* (Swinhoe). Physiol. Entomol..

[11] Dhillon MK, Tanwar AK, Hasan F (2019). Fitness consequences of delayed mating on reproductive performance of *Chilo partellus* (Swinhoe). J. Exp. Zool. A.

[12] Dhillon MK, Hasan F, Tanwar AK, Jaba J, Singh N, Sharma HC (2020). Genetic regulation of diapause and associated traits in *Chilo partellus* (Swinhoe). Sci. Rep..

[13] Chown SL, Terblanche JS (2006). Physiological diversity in insects: Ecological and evolutionary contexts. Adv. Insect Physiol..

[14] Sezonlin M, Dupas S, Le Rü B, Le Gall P, Moyal P, Calatayud PA, Giffard I, Faure N, Silvain JF (2006). Phylogeography and population genetics of the maize stalk borer *Busseola fusca* (Lepidoptera, Noctuidae) in sub-Saharan Africa. Mol. Ecol..

[15] Rowntree JK, Cameron DD, Preziosi RF (2011). Genetic variation changes the interactions between the parasitic plant-ecosystem engineer Rhinanthus and its hosts. Philos. Trans. R. Soc. B.

[16] Giron D, Dubreuil G, Bennett A, Dedeine F, Dicke M, Dyer LA, Kawakita A (2018). Promises and challenges in insect–plant interactions. Entomol. Exp. Appl..

[17] Williams RS, Howells JM (2018). Effects of intraspecific genetic variation and prior herbivory in an old-field plant on the abundance of the specialist aphid *Uroleucon nigrotuberculatum* (Hemiptera: Aphididae). Environ. Entomol..

[18] Matsubayashi KW, Ohshima I, Nosil P (2010). Ecological speciation in phytophagous insects. Entomol. Exp. Appl..

[19] Feder JL, Berlocher SH, Roethele JB, Dambroski H, Smith JJ, Perry WL, Gavrilovic V, Filchak KE, Rull J, Aluja M (2003). Allopatric genetic origins for sympatric host-plant shifts and race formation in *Rhagoletis*. Proc. Natl Acad. Sci USA.

[20] Althoff MD, Pellmyr O (2002). Examining genetic structure in bogus yucca moth: A sequential approach to phylogeography. Evolution.

[21] Knowles LL, Maddison WP (2002). Statistical phylogeography. Mol. Ecol..

[22] Templeton AR (2004). Statistical phylogeography: Methods of evaluating and minimizing inference errors. Mol. Ecol..

[23] Thomas Y, Bethenod MT, Pelozuelo L, Frérot B, Bourguet D (2003). Genetic isolation between two sympatric host-plant races of the European corn borer, *Ostrinia nubilalis* Hübner. I. Sex pheromone, moth emergence timing, and parasitism. Evolution.

[24] Sharma HC, Taneja SL, Kameswara Rao N, Prasada Rao KE (2003). Evaluation of sorghum germplasm for resistance to insect pests. Inf. Bull..

[25] Sharma HC, Dhillon MK, Pampapathy G, Reddy BVS (2007). Inheritance of resistance to spotted stem borer, *Chilo partellus* in sorghum, *Sorghum bicolor*. Euphytica.

[26] Kanta U, Dhillon BS, Sekhon SS, Mihm JA (1997). Evaluation and development of maize germplasm for resistance to spotted stem borer. Insect Resistant Maize: Recent Advances and Utilization.

[27] Rakshit S, Kaul J, Dass S, Singh R, Sekhar JC, Singh SB (2008). Catalogue of Indian maize inbred lines. Tech. Bull..

[28] Agrawal AA (2001). Phenotypic plasticity in the interactions and evolution of species. Science.

[29] Stireman JO, Nason JD, Heard SB (2005). Host-associated genetic differentiation in phytophagous insects: general phenomenon or isolated exceptions? Evidence from a goldenrod-insect community. Evolution.

[30] Zytynska SE, Preziosi RF (2011). Genetic interactions influence host preference and performance in a plant-insect system. Evol. Ecol..

[31] Zytynska SE, Preziosi RF (2013). Host preference of plant genotypes is altered by intraspecific competition in a phytophagous insect. Arthropod-Plant Interact..

[32] Sharma HC (2009). Biotechnological Approaches for Pest Management and Ecological Sustainability.

[33] Sharma HC, Dhillon MK, Hatfield JL, Sivakumar MVK, Prueger JH (2020). Climate change effects on arthropod diversity and its implications for pest management and sustainable crop production. Agroclimatology: Linking Agriculture to Climate.

[34] Smith CM (2005). Plant Resistance to Arthropods: Molecular and Conventional Approaches.

[35] Dhillon MK, Sharma HC (2012). Paradigm shifts in research on host plant resistance to insect pests. Indian J. Plant Protect..

[36] Funk DJ (1998). Isolating a role for natural selection in speciation: Host adaptation and sexual isolation in *Neochlamisus bebbianae* leaf beetles. Evolution.

[37] Dres M, Mallet J (2002). Host races in plant-feeding insects and their importance in sympatric speciation. Philos. Trans. R. Soc. B.

[38] Rundle HD, Nosil P (2005). Ecological speciation. Ecol. Lett..

[39] Schluter D (2009). Evidence for ecological speciation and its alternative. Science.

[40] Ishiguro N, Tsuchida K (2006). Polymorphic microsatellite loci for the rice stem borer, *Chilo suppressalis* (Walker) (Lepidoptera: Crambidae). Appl. Entomol. Zool..

[41] Mukhopadhyay J, Ghosh K, Rangel EF, Munstermann LE (1998). Genetic variability in biochemical characters of Brazilian field populations of the *Leishmania* vector, *Lutzomyia longipalpis* (Diptera: Psychodidae). Am. J. Trop. Med. Hyg..

[42] Vijaya Lakshmi P, Amudhan S, Bindu KH, Cheralu C, Bentur JS (2006). A new biotype of the Asian rice gall midge Orseolia oryzae (Diptera: Cecidomyiidae) characterized from the Warangal population in Andhra Pradesh, India. Int. J. Trop. Insect Sci..

[43] Himabindu K, Suneetha K, Sama VSAK, Bentur JS (2010). A new rice gall midge resistance gene in the breeding line CR57-MR1523, mapping with flanking markers and development of NILs. Euphytica.

[44] Ratcliffe RH, Cambron SE, Flanders KL, Bosque-Perez NA, Clement SL, Ohm HW (2000). Biotype composition of Hessian fly (Diptera: Cecidomyiidae) populations from the Southeastern, Midwestern, and Northwestern United States and virulence to resistance genes in wheat. J. Econ. Entomol..

[45] Zhou H, Wang X, Mo Y, Li Y, Yan L, Li Z, Shu W, Cheng L, Huang F, Qiu Y (2020). Genetic analysis and fine mapping of the gall midge resistance gene Gm5 in rice (*Oryza sativa* L.). Theor. Appl. Genet..

[46] Dhillon MK, Kumar S (2017). Amino acid profiling of *Sorghum bicolor* vis-à-vis *Chilo partellus* (Swinhoe) for biochemical interactions and plant resistance. Arthropod-Plant Interact..

[47] Dhillon MK, Kumar S (2020). Lipophilic profiling of *Sorghum bicolor* (L.) seedlings vis-à-vis *Chilo partellus* (Swinhoe) larvae reveals involvement of biomarkers in sorghum-stem borer interactions. Indian J. Exp. Biol..

[48] Atray I, Bentur JS, Nair S (2015). The Asian rice gall midge (*Orseolia oryzae*) mitogenome has evolved novel gene boundaries and tandem repeats that distinguish its biotypes. PLoS ONE.

[49] Fujita D, Kohli A, Horgan FG (2013). Rice resistance to planthoppers and leafhoppers. Crit. Rev. Plant Sci..

[50] Diehl RS, Bush GL (1984). An evolutionary and applied perspective of insect biotypes. Annu. Rev. Entomol..

[51] Claridge MF, Den Hollander J (1983). A biotype concept and its application to insect pests of agriculture. Crop Prot..

[52] Downie DA (2010). Baubles, bangles, and biotypes: A critical review of the use and abuse of the biotype concept. J. Insect Sci..

[53] Perring TM (2001). The *Bemisia tabaci* species complex. Crop Prot..

[54] Wenger JA, Michel AP (2013). Implementing an evolutionary framework for understanding genetic relationships of phenotypically defined insect biotypes in the invasive soybean aphid (*Aphis glycines*). Evol. Appl..

[55] Sharma HC, Taneja SL, Leuschner K, Nwanze KF (1992). Techniques to screen sorghum for resistance to insect pests. Inf. Bull..

[56] Kumar S, Dhillon MK (2015). Lipophilic metabolite profiling of maize and sorghum seeds and seedlings, and their pest spotted stem borer larvae: A standardized GC-MS based approach. Indian J. Exp. Biol..

[57] Dhillon MK, Kumar S, Gujar GT (2014). A common HPLC-PDA method for amino acid analysis in insects and plants. Indian J. Exp. Biol..

