# Supplementary material for: Biological and biochemical diversity in different biotypes of spotted stem borer, Chilo partellus (Swinhoe) in India
Source: Sci Rep. 2021 Mar 11;11:5735. doi: 10.1038/s41598-021-85457-2 (PMC7970982; doi:10.1038/s41598-021-85457-2)
Supplement: Supplementary file 1 — Supplementary Information [file 41598_2021_85457_MOESM1_ESM.docx]

**Biological and biochemical diversity in different biotypes of spotted stem borer, *Chilo partellus* (Swinhoe) in India**


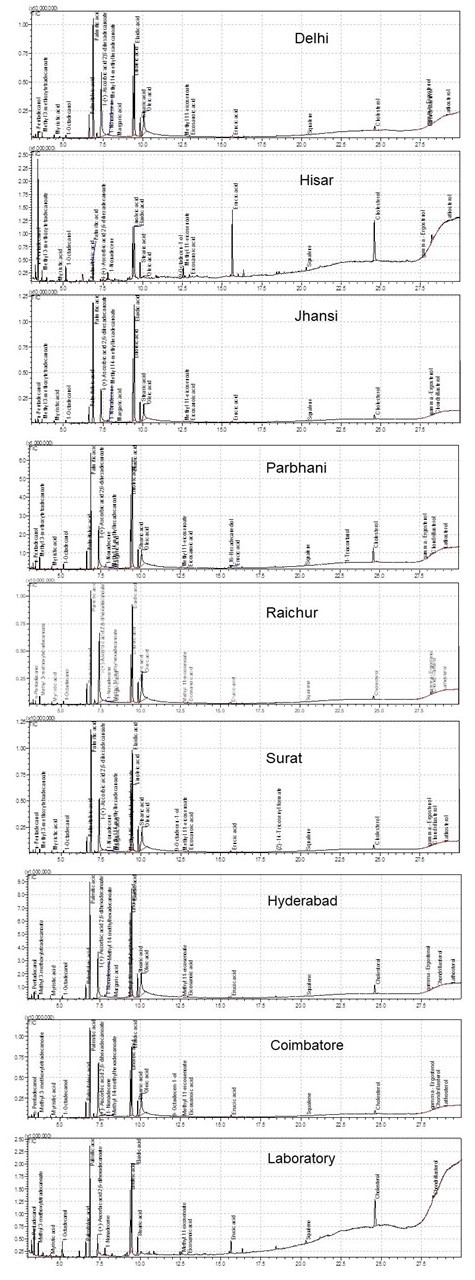
**Mukesh K. Dhillon, Aditya K. Tanwar, Sandeep Kumar, Fazil Hasan, Suraj Sharma, Jagdish Jaba and Hari C. Sharma**

Supplementary Fig. 1. Lipophilic profiles of different geographical *C. partellus* populations


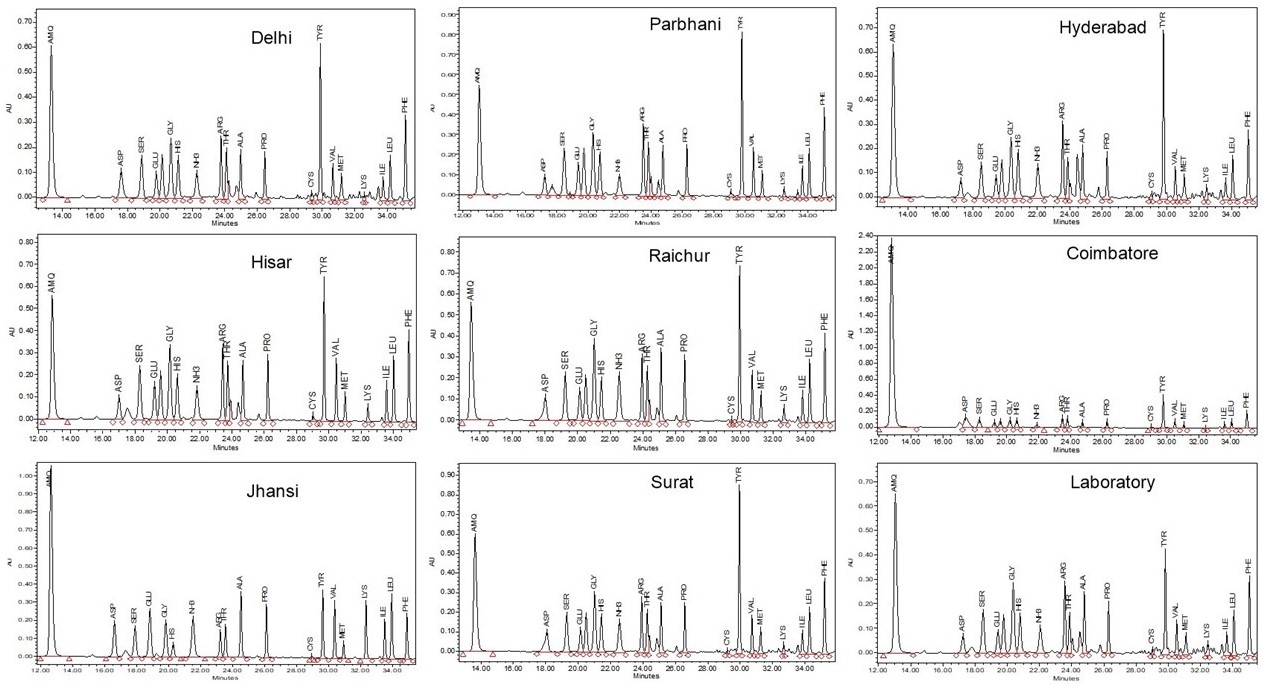


Supplementary Fig. 2. Amino acid profiles of different geographical *C. partellus* populations
